# AAV-mediated ERdj5 overexpression protects against P23H rhodopsin toxicity

**DOI:** 10.1093/hmg/ddaa049

**Published:** 2020-03-20

**Authors:** Monica Aguilà, James Bellingham, Dimitra Athanasiou, Dalila Bevilacqua, Yanai Duran, Ryea Maswood, David A Parfitt, Takao Iwawaki, Giannis Spyrou, Alexander J Smith, Robin R Ali, Michael E Cheetham

**Affiliations:** 1 UCL Institute of Ophthalmology, London EC1V 9EL, UK; 2 Division of Cell Medicine, Department of Life Science, Medical Research Institute, Kanazawa Medical University, Uchinada, 920-0293, Japan; 3 Department of Clinical and Experimental Medicine, Linköping University, Linköping, 581 83, Sweden

## Abstract

Rhodopsin misfolding caused by the P23H mutation is a major cause of autosomal dominant retinitis pigmentosa (adRP). To date, there are no effective treatments for adRP. The BiP co-chaperone and reductase ERdj5 (DNAJC10) is part of the endoplasmic reticulum (ER) quality control machinery, and previous studies have shown that overexpression of ERdj5 *in vitro* enhanced the degradation of P23H rhodopsin, whereas knockdown of ERdj5 increased P23H rhodopsin ER retention and aggregation. Here, we investigated the role of ERdj5 in photoreceptor homeostasis *in vivo* by using an *Erdj5* knockout mouse crossed with the P23H knock-in mouse and by adeno-associated viral (AAV) vector-mediated gene augmentation of ERdj5 in P23H-3 rats. Electroretinogram (ERG) and optical coherence tomography of *Erdj5*^−/−^ and *P23H*^+/−^:*Erdj5*^−/−^ mice showed no effect of ERdj5 ablation on retinal function or photoreceptor survival. Rhodopsin levels and localization were similar to those of control animals at a range of time points. By contrast, when AAV2/8-ERdj5-HA was subretinally injected into P23H-3 rats, analysis of the full-field ERG suggested that overexpression of ERdj5 reduced visual function loss 10 weeks post-injection (PI). This correlated with a significant preservation of photoreceptor cells at 4 and 10 weeks PI. Assessment of the outer nuclear layer (ONL) morphology showed preserved ONL thickness and reduced rhodopsin retention in the ONL in the injected superior retina. Overall, these data suggest that manipulation of the ER quality control and ER-associated degradation factors to promote mutant protein degradation could be beneficial for the treatment of adRP caused by mutant rhodopsin.

## Introduction

Retinitis pigmentosa (RP) is a group of retinal degenerative diseases that are characterized primarily by the loss of rod photoreceptor cells (1). RP is the most common cause of genetic blindness and affects approximately 1 in 3000 individuals in the United States (2). Individuals with RP experience night blindness, a progressive loss of peripheral visual fields and, eventually, loss of central vision as cone photoreceptor viability is compromised by rod cell death (3). Mutations in rhodopsin are a major cause of autosomal dominant RP, with over 200 mutations identified (4). In particular, the proline to histidine change at residue 23 (P23H) is a commonly studied mutation; indeed, it is the most common rhodopsin mutation found in North America (5). Rhodopsin mutations can be classified according to their molecular and cellular properties, and P23H belongs to the class II group of mutations, characterized by misfolding and intracellular inclusion formation (4,6). The P23 residue resides in the intradiscal domain of rhodopsin, and its substitution causes misfolding of the protein, reduces 11-*cis*-retinal binding and thus results in loss of function (7).

Misfolding of rhodopsin results in retention of the protein in the ER and subsequent degradation via the ubiquitin-proteasome system via ER-associated degradation (ERAD) (4). The folding and stability of rhodopsin are dependent on the formation of a disulfide bond between cysteine residues at positions 110 and 187 (C110 and C187) (8). Misfolded rod opsin can form a non-native disulfide bond between C185 and C187 (or C110 and C185), which traps rhodopsin in a misfolded state and causes loss of retinal binding (9).

Within the cell, networks of systems are responsible for maintaining correct protein function and homeostasis, known as proteostasis. Proteostasis mechanisms include systems for refolding, sequestering and degrading misfolded proteins, such as molecular chaperones, the unfolded protein response (UPR) and ERAD (10). Molecular chaperone proteins are important cellular factors for dealing with misfolded proteins. In particular, the ER resident HSP70 family member BiP (HSPA5) is involved in regulating the UPR and assisting the folding of ER synthesized proteins (11). The UPR helps to clear misfolded proteins by activating a transcriptional program that not only upregulates expression of molecular chaperones, degradation pathways and apoptosis mechanisms but also reduces the transcriptional load on the cell (12). Furthermore, BiP is involved in the normal biogenesis of rhodopsin, as well as the quality control of misfolded forms of rhodopsin (13), and BiP overexpression is protective in a P23H transgenic rat model (14). BiP function is regulated by ER-resident co-chaperones from the DNAJ family, of which there are several, including ERdj5 (15).

ERdj5 [also known as J-containing PDI-like protein (JPDI), or DNAJC10] is a multidomain protein containing a J-domain that interacts with BiP and six thioredoxin-like (TRX) domains. Of these TRX domains, four are active and contain CXXC motifs (16–18). ERdj5 has a role in ERAD for both glycosylated and non-glycosylated misfolded proteins and can act via its TRX domains as reductase for incorrect disulfide bonds in misfolded proteins, such as the one formed in misfolded P23H rhodopsin (19).

Another important component in rhodopsin proteostasis is the ER degradation-enhancing alpha-mannosidase-like 1 (EDEM1). EDEM1 enhances ERAD of terminally misfolded glycoproteins (20,21). We have previously shown that EDEM1 has dual activity towards mutant rhodopsin *in vitro* as it enhanced the degradation of P23H and also promoted its correct folding (22). Moreover, EDEM1 is expressed in photoreceptors and can be co-immunoprecipitated with wild-type (WT) rhodopsin from the ER (22). EDEM1 has also been shown to bind to cone opsin in 11-*cis*-retinal deficiency (23).

BiP and EDEM1 activity are coupled by ERdj5 to promote ER quality control and ERAD (18,24). The degradation of mutant rhodopsin *in vitro* by ERdj5 requires the combined activity of the J domain to recruit BiP and the thioreductase function (19), suggesting that ERdj5 could act as an important component of the ER quality control and degradation machinery in photoreceptors.

In this study, we investigated the effect of ERdj5 *in vivo* using an *Erdj5* knockout mouse (25). These animals are viable and healthy but show ER stress at sites of heavy protein secretion (e.g. salivary gland). Therefore, we also tested if *Erdj5* deletion might cause photoreceptor defects that would be more pronounced in the presence of proteotoxic stress from mutant rhodopsin. Furthermore, we tested whether increasing the levels of ERdj5 through gene augmentation could protect photoreceptors from P23H rhodopsin-mediated cell death.

## Results

### ERdj5 knockout mice do not show retinal degeneration

**Figure 1:**
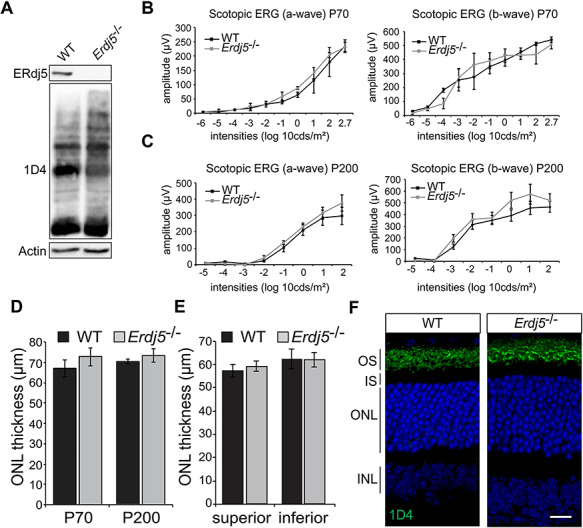
*Erdj5* knockout mice do not show retinal degeneration. (**A**) Western blot of WT and *Erdj5*^−/−^ retina lysate probed with ERdj5 and 1D4 anti-rhodopsin antibody. Actin immunoreactivity was used as a loading control. (**B**, **C**) Scotopic ERG responses, a- and b-wave of WT and *Erdj5*^−/−^ mice at (**B**) P70 (*n* = 4) and (**C**) P200 (*n* = 4). (**D**, **E**) ONL thickness measurements assessed by OCT of the total retina (**D**) and superior and inferior hemispheres (**E**). (**F**) Representative images of WT and *Erdj5*^−/−^ (P200) retina sections with rhodopsin stained in green and nuclei in blue with DAPI. Scale bars = 10 μm.

To examine the role of ERdj5 in photoreceptors, we investigated the retina of *Erdj5* knockout mice. ERdj5 was detectable by western blot in control, WT retina, but was absent in *Erdj5* knockout (*Erdj5*^−/−^), confirming that ERdj5 is normally present in retina and that the knockout lacks ERdj5 expression (Fig. 1A). Rhodopsin electrophoretic mobility and levels assessed by western blot were indistinguishable between *Erdj5*^−/−^ and WT animals (Fig. 1A). Photoreceptor function was assessed by a full-field scotopic electroretinogram (ERG) in dark-adapted mice at P70 and P200. Increasing light intensity correlates with a greater response of photoreceptor hyperpolarization (a-wave), followed by the propagation of the signal through the retina with subsequent depolarization (b-wave). *Erdj5*^−/−^ mice showed unaffected ERG responses, as both a-wave and b-wave response amplitudes were similar to those of the WT control mice at both time points (Fig. 1B and C). These data suggest that lack of ERdj5 does not impair visual function. Optical coherence tomography (OCT) was used to measure the thickness of the outer nuclear layer (ONL), as a marker of photoreceptor survival. The measurement of the ONL across the whole retina showed that *Erdj5*^−/−^ mice had the same ONL thickness as WT mice, suggesting the retina was healthy (Fig. 1D). There was also no difference in their ONL thickness in the superior or inferior retina (Fig. 1E). To confirm this observation, the retinae of *Erdj5*^−/−^ and WT mice were analyzed by histology (Fig. 1F). The ONL thickness and rhodopsin localization of *Erdj5^−/−^* mice were indistinguishable from the WT. Overall, no adverse effects of *Erdj5* knockout were observed in the retina.

### ERdj5 ablation in rhodopsin P23H mice does not affect retinal degeneration

**Figure 2:**
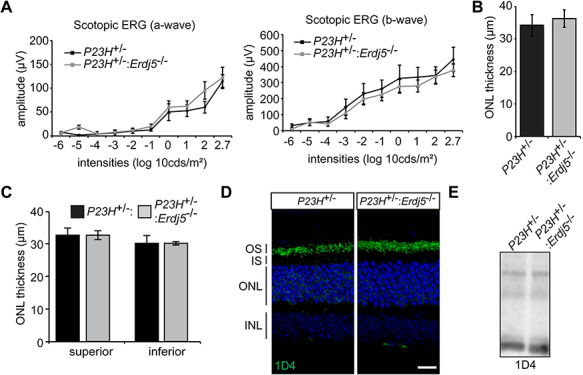
*Erdj5* ablation does not affect retinal degeneration in P23H rhodopsin mice. (**A**) Scotopic ERG responses of the *P23H*^+/−^:*Erdj5*^−/−^ animals compared to *P23H*^+/−^ (*n* = 4). (**B**, **C**) . ONL thickness measurements assessed by OCT for the total retina (**B**) and superior and inferior hemispheres (**C**). (**D**) Representative confocal images of *P23H*^+/−^ and *P23H*^+/−^:*Erdj5*^−/−^ retina showing no difference in retinal structure or rhodopsin localization between genotypes. Scale bars = 10 μm. (E) Western blot of the *P23H*^+/−^ and *P23H*^+/−^:*ERdj5*^−/−^ retinal lysates probed with the 1D4 antibody.

ERdj5 facilitates degradation of misfolded proteins via ERAD (26); therefore, ablation of *Erdj5* could exacerbate retinal degeneration in P23H rhodopsin mouse models. To determine the effect of lack of ERdj5, we crossed P23H knock-in (KI; *P23H*^+/−^) (27) mice with *Erdj5* knockout mice to produce *P23H*^+/−^:*Erdj5*^−/−^ and compared them with littermate *P23H*^+/−^:*Erdj5*^+/+^ animals. The *P23H^+/−^* mice have a moderate retinal degeneration phenotype with rod photoreceptor cell almost complete at 170 days of age (27). Therefore, we assessed visual function of the *P23H^+/−^:Erdj5^−/−^* animals at P70, a stage where the *P23H^+/−^* animals show clear rod cell dysfunction and death, but there are still photoreceptors remaining, enabling changes in the rate of disease in either direction to be observed. Scotopic ERG responses were similar in the presence and absence of ERdj5 (Fig. 2A). ONL thickness measurements assessed by OCT also revealed no difference, either in the total retina (Fig. 2B) or in the superior or inferior hemispheres (Fig. 2C). Immunohistochemistry of the *P23H*^+/−^:*Erdj5*^−/−^ retina showed no difference in retinal structure or rhodopsin localization to the P23H controls (Fig. 2D). The levels of rhodopsin expression assessed by western blot remained unchanged (Fig. 2E). These results suggest that loss of ERdj5 does not affect the photoreceptor response to misfolded rhodopsin.

### Subretinal delivery of AAV2/8-ERdj5 reduces the loss of P23H-3 rhodopsin photoreceptors

Previous *in vitro* data showed that overexpression of ERdj5 in cells could promote P23H rhodopsin degradation, reduce aggregation and inclusion formation (19). To investigate the effect of ERdj5 overexpression on P23H *in vivo*, we used adeno-associated viral (AAV)-mediated transduction of photoreceptors in the P23H-3 rat model. The rat model was chosen because the larger size of the rat eye facilitated subretinal injection, and this model has a well-characterized rate of degeneration similar to the P23H KI mouse model. P23H-3 rhodopsin rats received subretinal injections of either an AAV2/8 vector carrying ERdj5 with an HA tag (AAV2/8-ERdj5-HA) virus or PBS to the superior retina at P15. Following injection, we analyzed animals over 3 months by ERG and OCT and then euthanized them for histological and immunochemical analyses.

**Figure 3:**
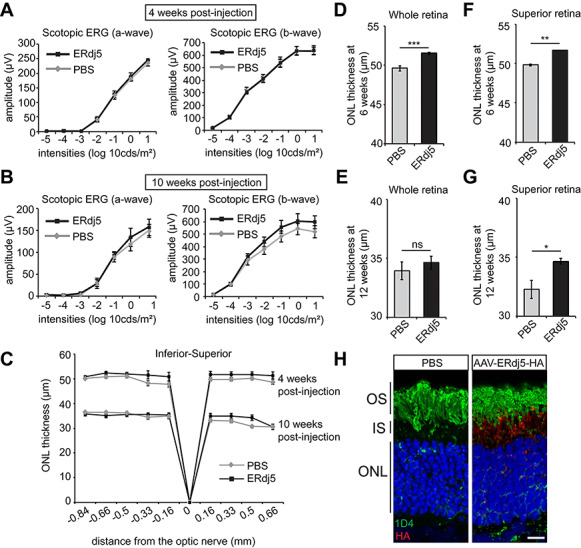
ERdj5 gene augmentation enhances photoreceptor survival in P23H-3 rats. (**A**, **B**) Scotopic ERG responses of P23H-3 rats subretinally injected with PBS or AAV-ERdj5-HA assessed 4 weeks (**A**) and 10 weeks PI (**B**) (*n* = 8). (**C**) Spider plot showing P23H-3 ONL thickness at 4 and 10 weeks after PBS or AAV-ERdj5-HA injection assessed by OCT (*n* = 8). (**D**–**G**) Mean ONL thickness across the whole retina (**D**, **F**) and the superior retina (subretinal injection site) (**E**, **G**) at 4 weeks (D, E) and 10 weeks (F, G) PI (*n* = 8). (**H**) Representative image of P23H-3 rat retina 4 weeks PI with PBS or AAV-ERdj5-HA at 6 weeks of age and stained with 1D4 antibody (green) and HA antibody (red). Scale bar = 10 μm. ns, not significant. ^*^P < 0.05; ^**^P < 0.01; ^***^P < 0.001.

Overexpression of ERdj5 in P23H-3 rats led to no difference in the ERG responses between the PBS- and the ERdj5-injected eyes 4 weeks PI (Fig. 3A). Ten weeks after injection, slightly better responses were observed in the eyes that had received ERdj5, but these did not reach statistical significance (Fig. 3B). OCT measurement 4 weeks PI for the AAV2/8-ERdj5-HA-injected eyes showed significant preservation of ONL thickness across the retina (Fig. 3D and E). At 10 weeks PI, there was not a significant change in ONL thickness in the treated eyes when measured across the whole retina. However, in the superior hemisphere of the retina, where the injection was performed, we observed a significant preservation of ONL thickness in the AAV2/8-ERdj5-HA-injected eyes compared to the PBS-injected retinae at both time points (Fig. 3F and G).

A strong HA signal was detected in the inner segment, where ERdj5 is expected to localize (Fig. 3H). The signal was restricted to the superior hemisphere of the retina where the subretinal injection was performed and was not detected in the inferior hemisphere. Histological analyses also confirmed that the AAV2/8-ERdj5-HA transduced retinae were thicker in the superior part, as they showed an extra photoreceptor nuclei layer compared to the PBS-injected retinae (Fig. 3H). These data suggest that Erdj5 overexpression is protective in P23H-3 rats.

### ERdj5 overexpression promotes rhodopsin degradation in P23H rats

**Figure 4:**
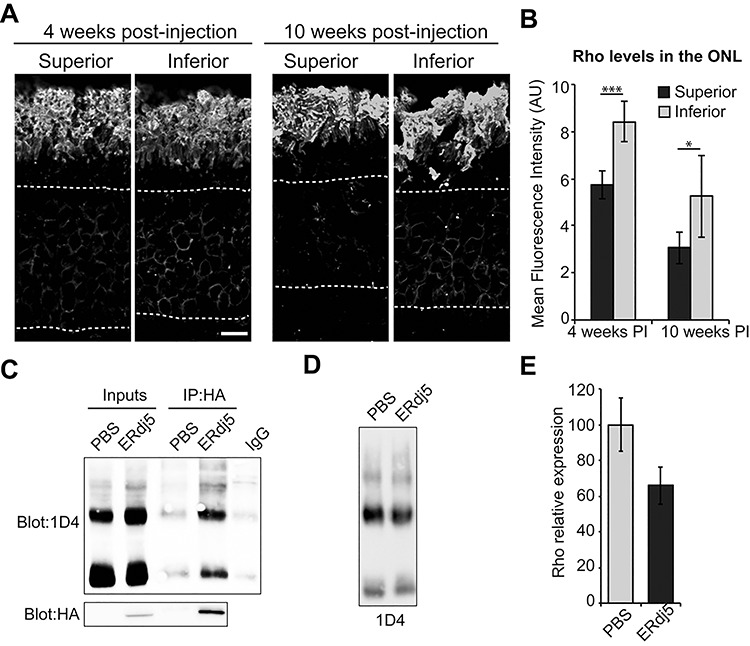
ERdj5 overexpression promotes rhodopsin degradation. (**A**) Representative images of the superior and inferior retinae of P23H-3 rats injected with AAV-ERdj5-HA and stained for rhodopsin at 4 and 10 weeks PI, 6 and 12 weeks of age, respectively. ONL marked with a dotted line. (**B**, **C**) Quantification of rhodopsin immunofluorescence intensity in the ONL showing increased levels of rhodopsin in the inferior retina in AAV-treated eyes (B), but not in control PBS-injected eyes (C) (*n* = 3). (**D**) Co-immunoprecipitation of rhodopsin with ERdj5, 4 weeks PI retinal lysates of PBS- or AAV-ERdj5-HA-injected eyes were incubated overnight with HA antibody (IP:HA) or control IgG (IgG), and immunopurified material was blotted with 1D4 antibody against rhodopsin. (**E**) Western blot of P23H-3 retinae injected with PBS or AAV-ERdj5-HA and probed with the 1D4 antibody. (**F**) Densitometric analysis was used to calculate the levels of rhodopsin in the AAV-ERdj5-HA-injected retinae relative to the PBS-injected (*n* = 4). ^*^P < 0.05; ^***^P < 0.001. Scale bar = 10 μm.

Immunohistological analysis of the treated retinae at 4 and 10 weeks PI revealed differences in rhodopsin localization between the superior and inferior retina (Fig. 4A). Quantification of fluorescence intensity in the ONL revealed a significant decrease in mislocalized rhodopsin in the superior retina, where there was a strong HA signal, at both time points compared to the inferior retina (Fig. 4B). This was in contrast to the PBS-injected retina, where the superior and inferior retinae were similar in intensity (Fig. 4C). To investigate whether ERdj5 could form a complex with rhodopsin, retinal lysates were co-immunoprecipitated with an antibody against the HA epitope and blotted with an antibody to rhodopsin. Rhodopsin was enriched when co-immunoprecipitated with ERdj5-HA in the AAV2/8-ERdj5-HA-injected eyes, whereas background levels of rhodopsin were detected in PBS-injected eyes with anti-HA, or with a control IgG immunopurification in AAV2/8-ERdj5-HA-treated retina (Fig. 4D). These data suggest that overexpressed ERdj5 can form a complex with rhodopsin *in vivo*. Retinal lysates of PBS- and AAV2/8-ERdj5-HA-injected eyes were blotted with 1D4 (Fig. 4E) and rhodopsin levels were quantified (Fig. 4F). ERdj5-injected retinae showed less rhodopsin compared to the PBS-injected retinas; however, this difference did not reach statistical significance. Collectively, these data suggest that ERdj5 overexpression can promote mutant rhodopsin clearance from the ER and enhance photoreceptor survival.

## Discussion

The results in this study suggest that ERdj5 is not essential for rhodopsin biogenesis or function *in vivo*. Loss of ERdj5 have did not have an effect on P23H rhodopsin expression, localization or pathogenicity either. This is in contrast to what was observed *in vitro*, where knockdown of ERdj5 affected the expression of both WT and P23H rod opsin in SK-N-SH neuroblastoma cells (19). The cause of this difference is not clear, but most likely relates to the heterologous overexpression system not fully reflecting the situation in photoreceptors. For example, heterologous rod opsin overexpression could affect ER protein loading, and unlike the retina the system has no 11-*cis*-retinal present, and retinoids are known to affect P23H folding (4). Similarly, the repertoire of ER chaperones in cultured cells might not accurately reflect the situation in photoreceptors. It has been suggested that different ER chaperones, such as PDI family proteins, other chaperones and DnaJ proteins, and ERdj5 have a redundant function in the ER protein quality control (28). This might explain why the lack of ERdj5 does not affect retinal degeneration, as other ER components are potentially compensating for its absence. Similarly, the photoreceptor ER quality control and ERAD machinery might be adapted to have less need for ERdj5.

By contrast, salivary gland function appears to require ERdj5, as ERdj5 ablation leads to a Sjögren’s syndrome-like phenotype in mice with induction of the UPR and the infiltration of immune cells, similar to that observed in patients (29). These data confirm the initial studies that the loss of ERdj5 principally affects the salivary gland (25) and is less critical in other tissues, where the requirement for coupling co-chaperone, reductase and ERAD for misfolded proteins is probably met by other factors.

Gene therapy approaches for dominant retinal disease, such as P23H rhodopsin RP, are more challenging than classical gene replacement/augmentation. Although gene augmentation with WT rod opsin has shown some benefit in P23H transgenic mice (30), supporting a dominant negative contribution to the pathogenesis, the majority of approaches have focused on either promoting photoreceptor survival in a gene-independent way or targeting the mutant allele. For example, allele-specific knockdown of the mutant transcript through ribozymes or antisense oligonucleotides (31,32), non-specific knockdown of both alleles with gene replacement (33,34) or CRISPR-based gene editing (35,36). Here, we show that viral-mediated overexpression of ERdj5 can improve photoreceptor viability in P23H rats. These results suggest that manipulation of ER quality control and ERAD factors could potentially benefit misfolding mutations in rhodopsin RP.

ERdj5 is a co-chaperone for BiP, which has also been suggested to play an important role in rhodopsin proteostasis (13,14). Indeed, overexpression of BiP also protects against P23H rhodopsin-mediated photoreceptor cell death (14). Studies of the P23H KI mouse have shown that over 90% of the mutant protein is degraded (27,37). Nevertheless, the P23H that escapes ER quality control and reaches the outer segment (OS) has detrimental effects on OS structure (27,38) and pharmacologically enhanced traffic of P23H rhodopsin to the OS accelerated retinal degeneration (39). Furthermore, expression of P23H rhodopsin has been suggested to induce ER stress (4). Whether this contributes to cell death is not certain, but the enhanced load on the photoreceptor proteostasis machinery and potential inhibition of protein degradation have also been implicated in photoreceptor cell death (4). Therefore, enhanced photoreceptor proteostasis and/or degradation of the mutant protein could promote photoreceptor survival.

Overexpression of ERdj5 led to reduced retention of rhodopsin in the ONL of P23H rat retina; this was only observed in the transduced areas of the retina and correlated with improved photoreceptor viability. There was a small reduction in the total amount of rhodopsin and a mild improvement in ERG, neither of which reached statistical significance. Full-field ERGs measure the response from the entire retina. Parallel injections with a AAV2/8:GFP indicated that photoreceptor transduction was also limited to the superior retina and approximately 50% of the photoreceptors in this region were transduced (data not shown), suggesting only 25% of total photoreceptors would be transduced, which might reduce the potential for an increase to a full-field ERG and limit any effect on total rhodopsin protein. The reduction in ONL staining for rhodopsin and total rhodopsin protein suggests improved clearance of rhodopsin from the ER. The ability of the transduced ERdj5-HA to bind rhodopsin suggests that this effect could be directly mediated by ERdj5 facilitating ERAD of the mutant protein.

It is important that any approach to enhance mutant rhodopsin degradation does not target the WT protein, as rod photoreceptors need sufficient rhodopsin to maintain viability (40). Unfortunately, we cannot discriminate between the WT and P23H protein in our assays as they only differ at a single residue, so we cannot determine if ERdj5 is specifically enhancing the clearance of the mutant protein or acting on WT rhodopsin, or even on other proteins. Nevertheless, *in vitro* studies showed that ERdj5 binds preferentially to P23H rod opsin (19), and in studies of other retinal disease genes, such as the R345W mutation in fibulin 3, ERdj5 also preferentially binds to the mutant protein (41). The correlation of the reduction in rhodopsin ER retention and improved cell viability support the notion that the mutant protein clearance is enhanced and other photoreceptor proteins, including WT rhodopsin, are not adversely affected.

These data support the hypothesis that enhancing the degradation of mutant rhodopsin through the delivery of important chaperones or ERAD factors could enhance photoreceptor survival in autosomal dominant retinitis pigmentosa. Previous studies of BiP gene delivery and proteasomal upregulation also support this concept (14,42). This approach could be used to complement other approaches that promote photoreceptor survival or target the mutant allele, such as allele-specific knockdown, to further lower the threshold of mutant protein expression and block toxic gain of function effects.

## Materials and Methods

### Animals

All animal procedures were conducted according to the Home Office (UK) regulations, under the Animals (Scientific Procedures) Act of 1986, and with the approval of the local UCL Institute of Ophthalmology, London, UK ethics committee. The P23H-3 line rats were kindly provided by Professor Matt LaVail (University of California San Francisco, USA, http://ophthalmology.ucsf.edu/wp-content/uploads/LaVail-RD-Rat-Model-Resource-063011.pdf) and crossed with WT Sprague-Dawley (SD) rats to generate heterozygous P23H-3 rats. SD rats were purchased from Harlan (Blackthorn, UK). *P23H* KI mice were generated as previously described (27). *Erdj5* knockout mice (*Erdj5^−/−^*) were generated as previously described (25). All animals were housed under a 12 h light (20–100 lux):12 h dark (<10 lux) cycle, with food and water available *ad libitum*. Both sexes were used evenly for experiments.

### Plasmid construct, production of AAV and intraocular administration

In order to preserve the inverted terminal repeat (ITR) sites of the pD10-CMV backbone yet create a more flexible multiple cloning site, the eGFP sequence was deleted from pEGFP-N1 vector (Clontech) by site-directed mutagenesis (NEB Q5 Site-Directed Mutagenesis Kit) using the following primers, delGFP_F: 5′-AAGCGGCCGCGACTCTAG-3′ and delGFP_R: 5′-CGACCGGTGGATCCCGGG-3′, to produce pCMV-delGFP. Subsequently, the multiple cloning site from pCMV-delGFP was excised with *NcoI* and *NotI* and cloned into the *NcoI* and *NotI* sites of pD10-CMV-GFP to create pD10-CMV-delGFP. The mouse ERdj5:HA:KDEL was excised from its pcDNA3.1(+) backbone (19,43) with *NheI* and *ApaI* and cloned into *NheI* and *ApaI* sites of pD10-CMV-delGFP to create pD10-CMV-ERdj5-HA-KDEL. NEB stable competent *Escherichia coli* cells were used throughout to protect the integrity of the ITRs.

Recombinant AAV2/8 serotype particles were produced through a previously described triple transient transfection method in HEK293T cells (44). AAV2/8 serotype particles were bound to an AVB Sepharose column (GE Healthcare) and eluted with 50 mm glycine pH 2.7 into 1 M Tris pH 8.8. Vector was washed in PBS and concentrated to a volume of 100–150 μl using Vivaspin 4 (10 kDa) concentrators. Viral genome titer was determined by quantitative real-time PCR using a probe-based assay binding the SV40 polyadenylation signal.

Viral vector administration was performed under general anesthesia using an operating microscope (Carl Zeiss, Cambridge, UK ) and a 34G needle (Hamilton). A total volume of 4 μl AAV2/8-Erdj5-HA was injected into P23H-3 rats at P15. Contralateral eyes were injected with PBS.

### Electroretinography

Animals were dark-adapted overnight in a ventilated light-tight box and anaesthetized with either ketamine/xylazine at 0.2 ml/100 g (rats) or with ketamine/domitor at 0.08 ml/10 g (mice) administered via an IP injection. Full-field scotopic ERG was carried out using the Diagnosys system and Espion software (Diagnosys, Lowell, MA) under red-light conditions, as previously described (39). Briefly, simultaneous bilateral recordings were taken using scotopic ERG protocols. Flash stimuli (10–1 ms duration, repetition rate 0.2 Hz) were presented via an LED stimulator (log intensity −5 to +1) under scotopic conditions. ERG responses were collected with the Espion software for analysis. Statistical analysis was performed using two-tailed Student’s *t*-test for samples with unequal variance.

### Optical coherence tomography

Mice were imaged using a Spectralis OCT as previously described (45). Rats were imaged using the Bioptigen Spectral-domain ophthalmic imaging system, as previously described (39). Briefly, image acquisition was obtained by using the rectangular scanning protocol consisting of a 2 mm × 2 mm perimeter with 750 A-scans (lines) and 5 B-scans (frames) with 60 frames/B-scan (for rat retina) or 1.4 mm × 1.4 mm perimeter with 750 A-scans (lines) and 10 B-scans (frames) with 20 frames/B-scan (for mouse retina). The Bioptigen InVivoVue Diver 2.0 was used to enable manual segmentation of the retinal layers and the ONL thickness was measured after exporting results from Diver to Excel. Statistical analysis was performed using two-tailed Student’s *t*-test for samples with unequal variance, except for the spider plots, which were analyzed by two-way analysis of variance with Sidak correction.

### Western blotting and immunoprecipitation

Retinae were extracted and lysed with RIPA buffer containing 2% (v/v) mammalian protease inhibitor cocktail. Retina lysates were sonicated for 2 × 15 s centrifuged for 15 min at 12 000×*g* at 4°C and diluted in sodium dodecyl sulfate (SDS) sample loading solution. Samples were resolved by SDS-polyacrylamide gel electrophoresis (SDS-PAGE) and western blotting. Immunodetection of rhodopsin was carried out with mouse anti-Rho-1D4 (1:1000; gift from Professor Robert Molday, Department of Biochemistry and Molecular Biology, University of British Columbia, Canada). Blots were probed with anti-mouse (1:50 000; 32 430, ThermoFisher Scientific Dartford, UK) secondary antibodies. Proteins were detected with the ECL Plus reagent (GE Healthcare). For immunoprecipitation, Protein G magnetic Dynabeads (Invitrogen, UK) were incubated with the retinae lysates and with 1–4 μg of specific antibody or 1–4 μg of non-specific IgG overnight at 4°C in an end-over-end rotator. Following incubation, the bead–antibody–protein complexes were washed four times with RIPA buffer. Samples were eluted from the beads with 30 μl of 2× SDS buffer, vortexed for 5 s and spun for 1 min at 12 000×*g*. Supernatant was collected via the Magnarak to minimize magnetic bead contamination. Samples were subjected to SDS-PAGE and western blotting.

### Immunohistochemistry

For fluorescence studies, eyes were collected at specified time points and fixed overnight in 4% paraformaldehyde at 4°C. Post-fixation eyes were cryoprotected by incubation in 30% sucrose in PBS. Eyes were then frozen and cryosectioned as previously described (45). Cryosectioned eyes were stained with mouse anti-Rho-1D4 (1:1500), mouse anti-HA (1:100, Sigma Aldrich Gillingham, Dorset, UK) primary antibodies in blocking solution (3% BSA, 10% normal goat serum) and visualized with Alexa Fluor 488 (1:1000; A11001/A11005, Life Technologies Ltd) and 594 (1:1000; A11007, Life Technologies Ltd Paisley, UK) conjugated IgGs. 4′,6-diamidino-2-phenylindole dihydrochloride (DAPI) (Sigma Aldrich) staining was used to visualize the nuclei.

### Fluorescence imaging and analysis

Retina images were obtained using a Carl Zeiss LSM700 laser-scanning confocal microscope. Images were exported with Zen 2009 software and were then prepared using Adobe Photoshop and Illustrator CS4. All measurements were performed in ImageJ (http://rsbweb.nih.gov/ij/). Statistical analysis was performed using two-tailed Student’s *t*-test for samples with unequal variance.
